# Estimating density limits for walking pedestrians keeping a safe interpersonal distancing

**DOI:** 10.1038/s41598-020-79454-0

**Published:** 2021-01-15

**Authors:** I. Echeverría-Huarte, A. Garcimartín, R. C. Hidalgo, C. Martín-Gómez, I. Zuriguel

**Affiliations:** 1 Departamento de Física y Matemática Aplicada, Facultad de Ciencias, Universidad de Navarra, Pamplona, Spain; 2 Department of Construction, Building Services and Structures, Universidad de Navarra, Pamplona, Spain

**Keywords:** Nonlinear phenomena, Statistical physics

## Abstract

With people trying to keep a safe distance from others due to the COVID-19 outbreak, the way in which pedestrians walk has completely changed since the pandemic broke out^1,2^. In this work, laboratory experiments demonstrate the effect of several variables—such as the pedestrian density, the walking speed and the prescribed safety distance—on the interpersonal distance established when people move within relatively dense crowds. Notably, we observe that the density should not be higher than 0.16 pedestrians per square meter (around 6 m^2^ per pedestrian) in order to guarantee an interpersonal distance of 1 m. Although the extrapolation of our findings to other more realistic scenarios is not straightforward, they can be used as a first approach to establish density restrictions in urban and architectonic spaces based on scientific evidence.

## Introduction

Among the eight preventive measures that were suggested by the World Health Organization (WHO) on 4th June 2020 to stop the COVID-19 spread^3^, the second and the third were (a) *to maintain at least 1 m distance with others*, and (b) *to avoid crowded places because there, it is difficult to maintain that 1 m distance*. Indeed, city authorities around the world are adopting different protocols to restrict the turnout at diverse venues such as nightclubs, markets, or even crowded open air places (streets, squares, beaches, and so on). In many cases, the imposed attendance restrictions are calculated from figures that were established based on fire risk regulations. The reason is that, despite the outstanding advances achieved in the field of pedestrian dynamics during the last 3 decades^4–13^, reliable information is lacking on very basic concepts that are crucial in the new pandemic scenario. For example, little is known about the interpersonal distance that people will be able to maintain with others when moving within a crowd of a given density. Also, the role of other variables that can affect the physical distance and the exposure times among pairs of individuals is poorly understood.

## The experiments

**Figure 1:**
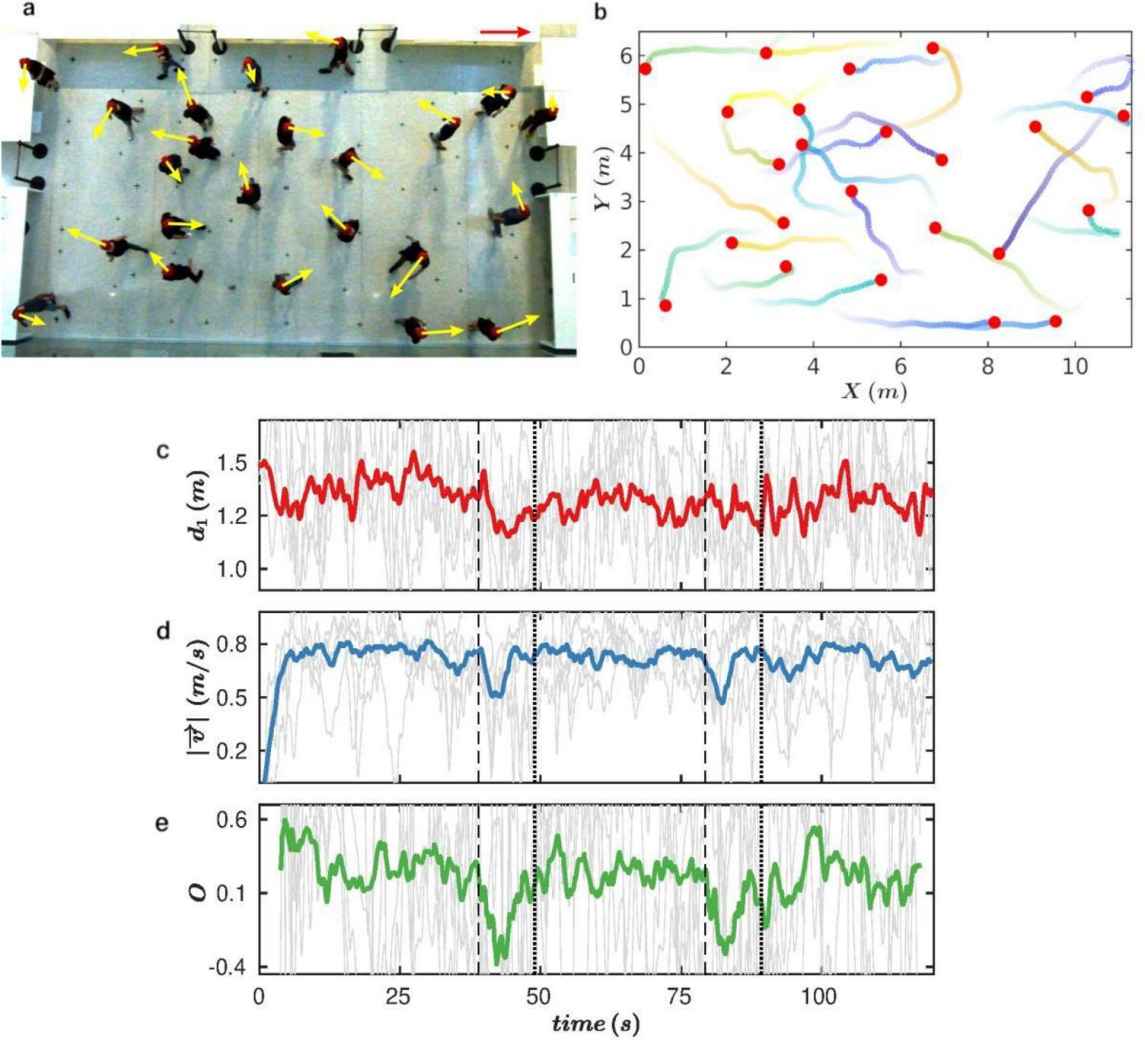
Experimental setup, pedestrian trajectories and time series. (**a**) Snapshot of an experiment with 24 people walking at a slow pace for a Prescribed Safety Distance (PSD) of 1.5 m. Yellow arrows correspond to the instantaneous velocities based on the scale indicated by the red vector (1 ms^−1^) at the right top of the panel. (**b**) Example of the trajectories followed by pedestrians during the 5 s previous to the snapshot shown in **a**. The colour of paths fades out with elapsed time (representation of the entire trajectories is given Fig. 5d, “Methods” section). (**c**–**e**) Temporal evolution of (**c**) the distances to the nearest pedestrian d1, (**d**) velocity modulus and (**e**) persistence in movement direction O for the same experiment shown in **a**, **b**. For each pedestrian, we calculated O as cos[θi(t + τ) − θi(t)] for a time delay of τ = 2 s, where θ(t) represents the direction of pedestrian velocity at time t, calculated as atan(Vy/Vx). Light grey lines represent the value of each magnitude for 5 chosen pedestrians, whereas solid coloured lines represent the value of the variable averaged over the total number of pedestrians in the experiment (24 in this case). Vertical dashed and dotted lines indicate the times at which the change in the movement direction start and finish, respectively.

In this work, we aim to fill this gap by implementing controlled laboratory experiments in which a number of volunteers were asked to roam inside a 76 m^2^ area while keeping a prescribed safety distance (PSD). This was done under different conditions of crowd density (ρ) and walking speed (WS). We performed 24 runs in 12 different experimental conditions (each scenario was repeated twice to check for consistency) which are summarized in Table 1. The evaluated conditions were: slow and fast walking speed, prescribed safety distance of either 1.5 or 2 m, and ρ = 0.16, 0.24, 0.32 and 0.42 pedestrians per square meter; corresponding to 12, 18, 24 and 32 volunteers in the arena respectively. Each run was conducted as follows: first, keeping a distance among each other above 2 m, the volunteers entered the enclosure, which was 11.4 m long by 6.7 m wide and was delimited by 90 cm high tables (Fig. 1a). Once within the room, pedestrians placed themselves (with no indication about their facing direction) at spots marked with a cross on the floor at 2 or 1.5 m depending on the prescribed safety distance requested in each experiment. Then, a whistle indicated the volunteers to start walking. After approximately 40 s, another audible signal was given and the participants had to walk towards any of the 4 walls, touch it (at any place) and resume the movement. The same scheme was repeated after another 40 s (or, 80 s from the beginning of the run). After 2 min of walking the experiment was called to an end; then all participants had to exit the room (keeping the prescribed safety distance) through one of the four exits and return to their seats (see “Methods” section for a more detailed description of the experiments and instructions given to pedestrians).

**Table 1:** Summary of experiments.

No.	Pedestrians^a^	ρ (ped m^−2^)	PSD (m)	WS	Reps^a^
1	12	0.16	2	Slow	2
2	12	0.16	2	Fast	2
3	18	0.24	2	Slow	2
4	18	0.24	2	Fast	2
5	18	0.24	1.5	Slow	2
6	18	0.24	1.5	Fast	2
7	24	0.32	2	Slow	1
8	24	0.32	2	Fast	1
9	24	0.32	1.5	Slow	2
10	24	0.32	1.5	Fast	2
11	32	0.42	1.5	Slow	2
12	32	0.42	1.5	Fast	2

^a^Pedestrians: Number of pedestrians within the arena. ρ: Average density values measured inside the 76 m^2^ area. PSD: Prescribed safety distance that pedestrians were asked to keep. WS: Walking speed (prescribed). Reps: Number of repetitions performed for each experiment.

All tests were registered with a video camera hanged at 12 m above the floor (see “Methods” section). From these videos, the positions of all participants were obtained at each frame (every 0.04 s). Then, we computed the trajectories (Figs. 1b, 5d) and other derived magnitudes that have become relevant due to the pandemic: first, d_1_, the distance of each participant to the nearest neighbour (i.e. the closest person); second, v, the velocity of each individual (see vectors in Fig. 1a); third, t_exp_, the time that two pedestrians spend at a distance smaller than 1.5 m uninterruptedly; and fourth, the persistence of the movement direction O, defined for each pedestrian i as O = cos[θ_i_(t + τ) – θ_i_(t)] where θ_i_(t) is the pedestrian’s velocity direction at time t. Therefore, O characterizes the change in the direction of motion that each pedestrian makes during a time lapse τ starting from t.

## Results

In Fig. 1c, d, e the temporal evolution of d_1_, v and O are displayed for a single run. Clearly, the values of these parameters for each individual are strongly fluctuating (grey lines), a behaviour that persists (although minimized) when representing the average of all the individuals in the arena at each time. Interestingly, the instants at which the volunteers were asked to move towards any of the walls (marked by the two vertical dashed lines in Fig. 1c, d, e) evidence a characteristic feature in the graphs as the magnitudes of both v and O reduce significantly before relaxing again to the values previous to the signal. This behaviour can be easily explained: when hearing the signal, in order to move towards the corresponding wall, pedestrians reassessed their course (so velocity reduced) and changed their movement direction (O also decreased). In this work we are mostly interested in the random movement, so from now on the analysis will be performed excluding the 10 s after the pedestrians start moving towards the wall (the intervals between dashed and dotted vertical lines). We have therefore about 200 s of data for each condition as two runs are carried out for each one. Although the d_1_ signal (Fig. 1c) suggests that there is not a significant effect of the change of direction on this quantity, for coherence, we will also exclude the same time windows as for the other variables.

**Figure 2:**
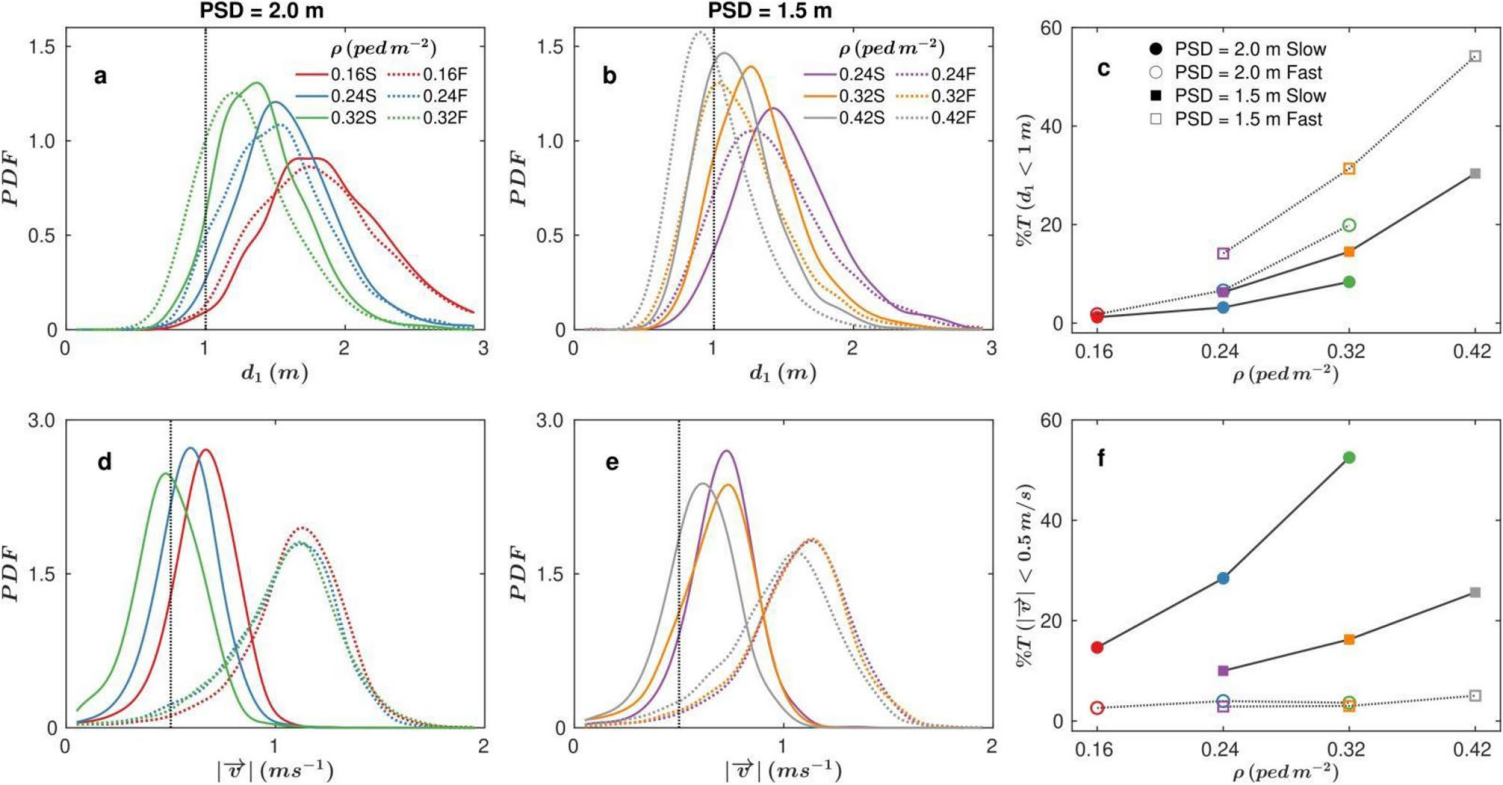
Minimum distances to the nearest neighbour and pedestrian velocities. (**a, b**) Probability density functions (PDFs) of the distances to the nearest pedestrian (d_1_) for a Prescribed Safety Distance (PSD) of (**a**) 2 m and (**b**) 1.5 m for different crowd densities, as indicated in the legend. Solid lines correspond to slow walking speed (S in the legend) and dotted lines to fast walking speed (F in the legend). (**c**) percentage of time that, on average, a pedestrian has a neighbour closer than 1 m, as a function of the crowd density for the different experimental conditions explored (see legend). (**d, e**) PDFs of velocity modules for different densities for (**d**) PSD = 2 m and (**e**) PSD = 1.5 m. Same legend as in (**a, b**). (**f**) Percentage of time that the velocity of a pedestrian is below 0.5 m/s for different PSD and walking speed values. Same legend as (**c**). Vertical dashed line in (**a**, **b**, **d**, **e**) provide a reference to help in the interpretation of figures (**c**, **f**).

Due to its significance in COVID-19 spread—according to the WHO^3^—we first focus our analysis on d_1_, which is the distance from each pedestrian to the closest neighbour. In Fig. 2a we present the distributions of this variable when the prescribed safety distance (PSD) is 2 m, for different crowd densities (ρ) and walking speeds (WS). Remarkably, even for the lowest density (0.16 ped m^-2^), more than 50% of the d_1_ data fall below 2 m; hence implying that—on average—each pedestrian has a neighbour closer than 2 m more than 50% of the time (note that the sampling is regular in time every 0.04 s). In addition, raising the density of pedestrians increases the number of times during which the prescribed safety distance is violated; indeed, for 0.32 ped m^−2^ almost everyone infringes the rule all the time. Also, increasing the walking speed leads to a weak displacement of the distributions towards the left, with the exception of the lowest density case. Therefore, we conclude that augmenting walking speed is prejudicial to keep the prescribed safety distance in dense scenarios, an issue that may be related to the increase of the travelled distance during the reaction time when the walking speed is faster. Similar behaviour occurs when the prescribed safety distance is 1.5 m (Fig. 2b), where the distributions also shift towards smaller values of d_1_ as ρ and walking speed increase. Next, in order to compare all the explored scenarios and to assess quantitatively the number of very close approaches, we have represented in Fig. 2c the percentage of time during which pedestrians have their first neighbour closer than 1 m. As said before, the crowd density primarily determines the established interpersonal distance suggesting that only densities as small as 0.16 ped m^-2^ can be considered “safe”. The different curves represented in Fig. 2c also evidence the convenience of enforcing long safety distances: the percentage of time in which pedestrians are closer than 1 m is consistently smaller when setting 2 m (circles) than 1.5 m (squares).

In Fig. 2d, e we display the distributions of pedestrian velocities, which demonstrate that volunteers did follow the instructions concerning the walking speed: the scenarios of fast walking speed display consistently higher values than those with slow ones. Also, our measurements reveal that the “fast speed” condition was, indeed, “normal speed” as an average speed of 1.2 m s^−2^ cannot be actually considered “fast”. Having said that, we will keep calling this scenario “fast speed” for coherence with the indications given to the participants. Interestingly, it seems that for slow walking speed the increase of density leads to a reduction of pedestrians’ speeds, a feature that is barely appreciated for high walking speeds. Aiming to quantify the number of times that people stop, we compute the proportion of velocities below 0.5 ms^−1^ (Fig. 2f). This plot shows that, when asked to move fast, volunteers never stop; a behaviour that could explain the augment of number of times in which the prescribed safety distance is violated, as mentioned before. On the contrary, when people move slowly, the number of stops is significant and increases when the prescribed safety distance was 2 m. This later dependence is probably due to the higher difficulty that pedestrians face to maintain this safety distance, which would make them stop more frequently than when it is 1.5 m.

**Figure 3:**
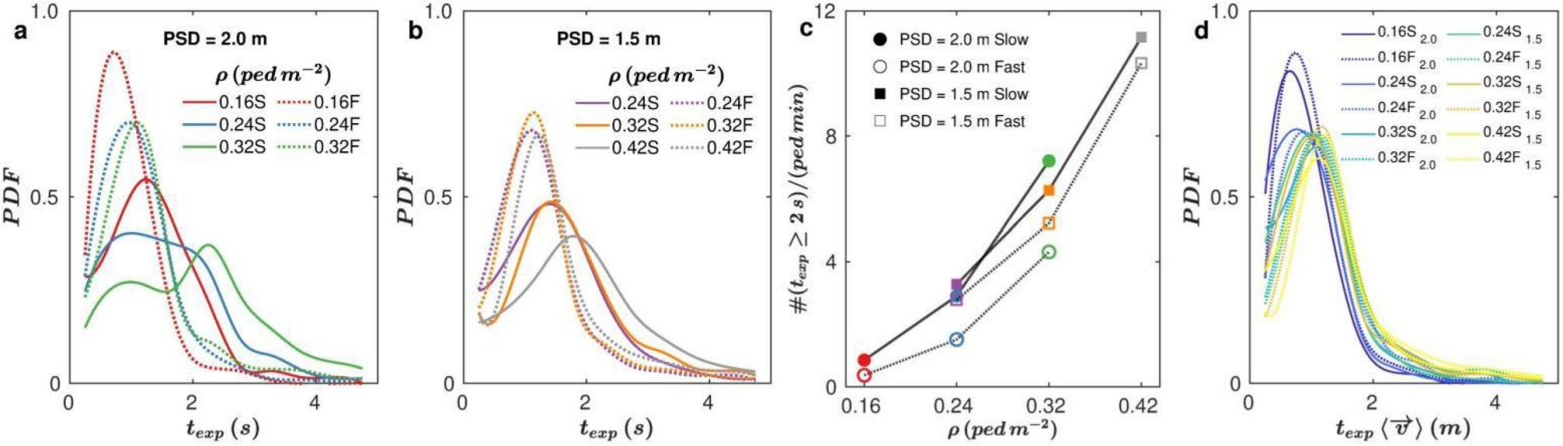
Exposure times and persistence of movement direction. (**a, b**) Distributions of exposure time periods (t_exp_) during which two pedestrians are uninterruptedly closer than 1.5 m. Probability density functions (PDFs) of t_exp_ when the prescribed safety distance is (**a**) 2 m and (**b**) 1.5 m for different densities, as indicated in the legend. Solid lines correspond to slow walking speed (S in the legend) and dotted lines to fast walking speed (F in the legend). (**c**) Number of times that a pedestrian is uninterruptedly closer than 1.5 m to another during a t_exp_ greater or equal to 2 s, as a function of density, for the experimental conditions indicated in the legend. The number of events recorded in each condition is normalized by the number of pedestrians within the room and the total duration (in minutes) of the test. (**d**) PDFs of t_exp_ re-scaled by the pedestrian average speed modulus measured at each experiment. Colours are the same for experiments with the same density; solid lines correspond to slow walking speed and dotted lines to fast walking speed.

Another magnitude that is considered to be related to the COVID-19 spread is the time that a pair of pedestrians remain at a potentially dangerous distance. Although this distance is still not strictly known, we have chosen 1.5 m and computed the corresponding exposure times, t_exp_, as the time lapses during which a pair of pedestrians remain closer than this distance uninterruptedly. Note that this definition of “exposure time” is only one among some others that are used in the framework of COVID-19^13^. As expected, the distributions of t_exp_ (Fig. 3a, b) display a shift towards higher values when the density increases. Nevertheless, and opposite to the behaviour observed for the distributions of d_1_, the exposure times are systematically higher for low walking speed. In order to quantify this effect we have computed the number of events in which t_exp_ is above 2 s. This magnitude—normalized by the number of pedestrians in the arena and per minute of test—is plotted versus ρ for all the scenarios investigated (Fig. 3c) demonstrating, once more, the prejudicial role of increasing the density above 0.16 ped s^−1^. Also, the higher values of t_exp_ emerging in the slow speed conditions are confirmed, demonstrating the two-sided effect of increasing the walking speed: on one hand the number of times that the prescribed safety distance is violated increases with walking speed (Fig. 2a, b); but on the other hand the exposure times are reduced. In Fig. 3d we have represented the same distributions of t_exp_ than in Fig. 3a, b but multiplying the exposure times by the average velocity of pedestrians in each case. With the sole exception of the runs at ρ = 0.16 ped m^−2^, a nice collapse is obtained for all curves which, incidentally, display a sharp peak at about 1 m. We speculate that this distance is the one that a pedestrian needs to walk before solving the conflict of a close approach and regain a safe interpersonal distance. Of course, this value is related to the arbitrary choice of 1.5 m as a dangerous distance, and may be also affected by the individual reaction time.

**Figure 4:**
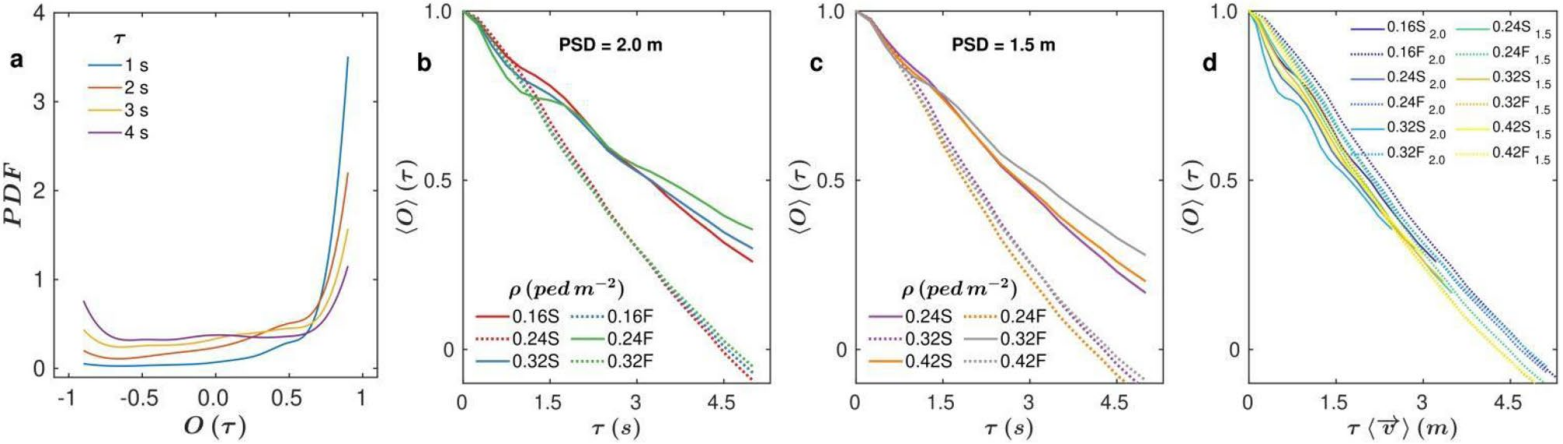
Persistence of movement direction. (**a**–**c**) Probability density functions (PDFs) of the persistence of movement direction O for an experiment in which ρ = 0.24 ped m^−2^, the prescribed safety distance (PSD) was 2 m, and the walking speed was fast (see Number 4 in Table 1). Different colours are used to represent distributions with different values of τ as indicated in the legend. (**b, c**) ‹O› as a function of τ for (**b**) PSD = 2 m and (**c**) PSD = 1.5 m for different densities values as indicated in the legend. (**d**) ‹O› as a function of the selected τ value re-scaled by the average speed modulus measured for each experimental condition. Solid lines correspond to slow walking speed (WS) and dotted lines to fast walking speed.

Finally, we examine the persistence of the movement direction and its dependence on the different parameters (Fig. 4a–d). First, in Fig. 4a we show the distributions of O obtained for different values of τ in the scenario of ρ = 0.24 ped m^−2^, a prescribed safety distance of 2 m and fast walking speed (experiment 4 in Table 1). For τ = 1 s, the distribution shows a sharp peak at O = 1 revealing that most pedestrians have not changed their moving direction after 1 s. As τ grows, the distributions widen and a small peak at O = − 1 starts emerging, indicating that a number of pedestrians have inverted their movement direction (θ(t + τ) − θ(t) = π). Finally, for τ = 4 s a bimodal distribution is observed with peaks at O = 1 and O = − 1, that corresponds to a situation in which most of pedestrians either keep their moving direction or have inverted it.

Aiming for a more complete characterization of this phenomenon, the average of these distributions was calculated for different values of the time lag τ. These temporal correlations of the movement directions are presented in Fig. 4b, c evidencing that basically, the only parameter that affects them is the walking speed: fast speeds lead to quicker decays than slow ones. Thus, all data can be rescaled multiplying τ by the average velocity of the volunteers in each case (Fig. 4d). Interestingly, all curves collapse reaching ‹O› = 0 (50% of the people have reversed their course) for a characteristic length of about 4.5 m, which is about half of the room size. This suggests that people, when moving inside the arena, mainly follow a fixed direction until they approach to any of the walls. The fact that all curves collapse independently of ρ, the walking speed and the prescribed safety distance, also indicates that people avoid collisions with others by just slightly modifying the movement direction (and not turning around, for example). Incidentally, this is the expected behaviour in real life.

As a final remark, let us stress once more that in idealistic laboratory tests, density crowds above 0.16 ped m^−2^ do not allow people to move keeping a safe interpersonal distance of 1 m as requested by the WHO. Of course, this value should be taken with care as, in real life, it would depend on the specific spatial arrangement, as well as on a number of variables related to human behavior (walking in groups, stationary standing people waiting for someone…). Anyway, in order to contextualise the figure of 0.16 ped m^−2^, let us consider the case of a mall where the results of our experiment could be extrapolated (people who look at products are brought together with others who have a fixed objective). Although each country has its own regulations, and the adaptation to the pandemic situation has been different, based on Spanish regulations^20^ the capacity of malls in the most restrictive scenario has been precisely 0.17 ped m^−2^ (one third of the 0.5 ped m^−2^ allowed in normal conditions). Importantly, this capacity is calculated considering the whole surface of the mall without any consideration on the possibility of people concentrating at specific locations.

Besides determining the dangerous density of 0.16 ped m^−2^ in our idealistic experimental conditions, we evidence the importance of prescribing a considerable safety distance as some people may likely underestimate the actual value of the interpersonal distance. This would be the reason for which the number of approaches shorter than 1 m is more important for the 1.5 m prescribed distance. Again, although this finding could be considered by authorities, it should be taken with due caution as there are other factors that may significantly alter the perceived safe interpersonal distance.

Apart from the limited, but promising, impact of this work to help set the occupancy limits in urban or architectonic spaces, we believe that our experimental data can be used to validate new pedestrian models^14^; or to readjust previous ones^15,16^ in which the accepted distances among pedestrians were much shorter. Undoubtedly, the COVID-19 outbreak has changed human behaviour and this necessarily affects the way people walk. Therefore, a new or readapted framework is necessary in the field of pedestrian dynamics as some magnitudes (such as the distance to the closest neighbour) and variables (such as the prescribed safety distance) have acquired a remarkable relevance. Finally, let us also state that our findings could be integrated in large-scale epidemiologic models^17–19^ giving rise to more precise predictions of virus spreading.

## Methods

A total of 38 volunteers (28 men and 10 women) participated in the experiment, which took place in a university building on 23 June 2020 and was conducted for 3 h approximately. All the persons performing these exercises gave informed consent concerning their participation in the drills. In addition, all methods were carried out in accordance with the guidelines and regulations applying at the day of the experiment in the Region of Navarra (Spain) and the experimental protocols were approved by the University of Navarra ethics committee.

**Figure 5:**
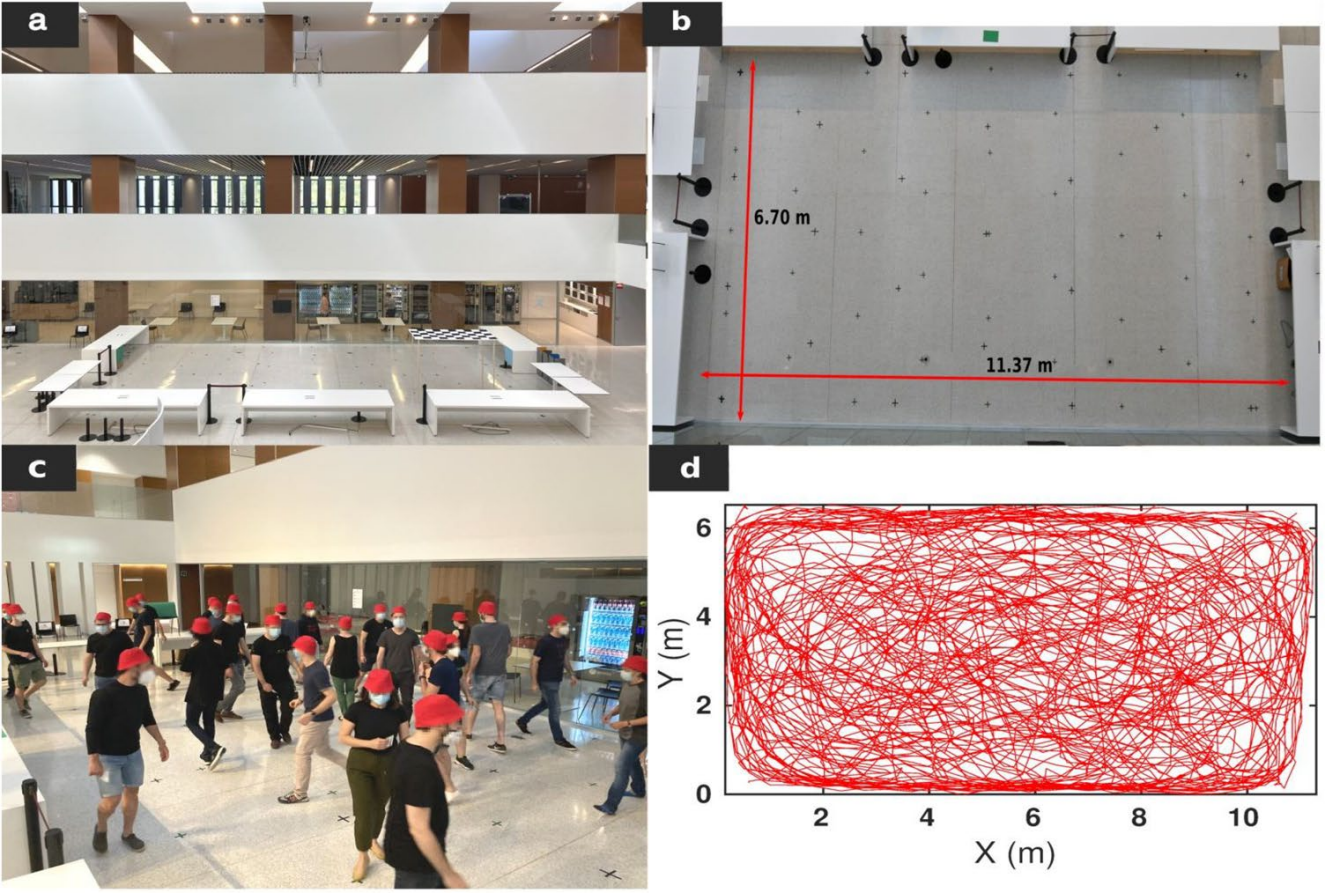
(Methods) | Experimental scenario. (**a**) Side view of the arena delimited by several tables. In the far right corner of the arena the reference checkboard (1.8 m height) can be seen. At the top of the picture, the camera is hanging at around 12 m. (**b**) Top view of the experimental scenario delimited by tables. On the floor, the different crosses marking the initial positions for the experiments with a prescribed distance of 2 m (black spots) and 1.5 m (green spots) can be observed. (**c**) Picture in which pedestrians wearing dark clothes, masks, and a red hat, are participating in one of the drills. (**d**) Set of pedestrians’ trajectories for the same experiment as in Fig. 1 (24 people walking at a slow pace for a prescribed safety distance of 1.5 m). Horizontal and vertical axes label the distance from the bottom-left corner of the scene, in meters.

The participants wore dark clothes and each one was given a red hat to speed up the subsequent image processing (Fig. 5c). In addition, each participant had an ID-Card with a number and a unique combination of 4 colours and letters (A, B, C and D). Furthermore, given the sanitary circumstances, the volunteers were required to wear a mask during all the experiment. When arriving to the building, the volunteers were asked to sit on the chair labelled with the number of their ID-card. These chairs were conveniently placed near the arena with a distance larger than 2 m among them. In each test, a number of participants were required to enter the arena over the loudspeaker (by means of the number assigned on their ID-card). The arena was 11.37 m long by 6.70 m width and was delimited by tables of 90 cm high leaving space for 4 doors (one on each of the short sides and two on one of the long sides) as illustrated in Fig. 5a, b. Once inside and depending on the prescribed safety distance (PSD) suggested, they had to take as their starting position one of the positions marked with a cross on the ground (see spots in Fig. 5b) leaving their initial orientation free to choose. These marks, previously delimited at 2 m or 1.5 m (depending on the PSD value), served as a reference to the participants to estimate the distance that they had to respect; also this strategy allowed us to make sure that all the participants initially respected the prescribed safety distance. Once everybody had reached their positions, they were informed of the walking speed (WS) that they had to maintain depending on the experimental condition (no indication was given about whether they could stop or not to avoid conflicts). As an example of slow walking speed, they were put in the situation of window shopping. For the fast case, they were left to give themselves an interpretation of what they considered fast, but it was recalled to them that their priority should always be trying to respect the safety distance.

Once the instructions had been given, a sound signal started the experiment. Initially, all pedestrians could walk freely for around 40 s. This allowed pedestrians to roam the arena casually (at random), always trying to respect the prescribed safety distance to the rest of the people. Once the time was up, an indication was given and the pedestrians had to go to one of the four walls of the enclosure, touch it at any point and walk freely again. In order to avoid that all pedestrians go to their nearest wall at the moment of the signal, a letter was announced through the loudspeaker, then each pedestrian had to move to the wall identified with a big card of the colour that corresponded to that letter in his/her ID card. In this way the number of people going to each of the 4 walls was approximately the same. Again, after another 45 s of free movement, the same strategy was repeated. Finally, after 2 min, an ending signal indicated pedestrians that they should leave the arena (keeping the prescribed safety distance) through one of the 4 doors that was opened randomly for each experiment. Once outside, each person had to sit in their individual chair and rest until being required again.

Every experiment was recorded by a camera at 25 fps with a 4 K (i.e., 3840 × 2160 pixels) resolution. It was hanged at 12 m. from the floor pointing downwards (Fig. 5a) with a small inclination, and with an aspherical objective to avoid distortion. In order to correct the image deformation due to camera tilt (distortion was found to be negligible) the positions were calibrated taking around 40 pictures of a reference checkboard placed at around 1.8 m height (Fig. 5a). Then, every participant’s red hat was tracked along time by means of a software written by us. It involved colour segmentation to identify hats and finding their centroids at every frame. In this way, we got the position of every participant with an estimated absolute precision of 10 cm (approximately, the size of dots in Fig. 1b). In this way we obtained the trajectories of each pedestrian inside the arena over the whole experimental realization. As can be seen in Fig. 5d, pedestrians occupied more or less homogeneously the whole arena, although there is a slightly higher concentration of individuals walking parallel to the walls. The instantaneous velocities for each person were calculated directly from the positions using a time interval of 0.76 s to reduce spurious noise. The obtained results were quite insensitive to the choice of this parameter (we tested 0.64 s and 1 s without finding significant differences). Then, from the velocity vectors for each pedestrian, we defined their velocity direction θ as θ = atan(V_y_/V_x_). After this analysis, for each video we obtain the list of position, speed and movement direction of each pedestrian at all times.

The first magnitude we defined was the distance to the first neighbour d_1_. Knowing the positions of each pedestrian, we calculated the modulus of the distance of each pedestrian to the rest and we took the minimum of these values.

$${\varvec{d}}_{1} = {\varvec{min}}\left( {\left| {\overrightarrow {{{\varvec{d}}_{{{\varvec{ij}}}} }} } \right|} \right)\user2{ }\forall \user2{ i},\user2{j i} \ne {\varvec{j}}$$

In order to know how the direction of the pedestrians’ movement changed during the experiment, we introduced O which we defined as the persistence in velocity direction. This magnitude is defined for each pedestrian as

$${\varvec{O}}\left( {{\varvec{t}},{\varvec{\tau}}} \right) = \user2{ }\cos \left( {{\varvec{\theta}}\left( {{\varvec{t}} + {\varvec{\tau}}} \right) - {\varvec{\theta}}\left( {\varvec{t}} \right)} \right)$$

where θ(t) is the orientation of the velocity vector of each pedestrian at a given time and τ is the time lapse in which O is calculated. This quantity tends to 1 if pedestrians do not change their movement direction and − 1 if they completely change it (they move in the opposite direction).

Finally, we calculated the exposure time t_exp_ during which two pedestrians were below 1.5 m distance uninterruptedly as

$${\varvec{t}}_{{{\varvec{exp}}}}^{{{\varvec{ij}}}} = \user2{ }\mathop \sum \limits_{{\varvec{t}}} {\varvec{h}}\left( {{\varvec{i}},{\varvec{j}},{\varvec{t}}} \right)$$

With

$${\varvec{h}}\left( {{\varvec{i}},{\varvec{j}},{\varvec{t}}} \right)\left\{ {\begin{array}{*{20}l} 1 \hfill & {if\quad \left| {\overrightarrow {{{\varvec{d}}_{{{\varvec{ij}}}} }} \left( {\varvec{t}} \right)} \right| \le 1.5 m} \hfill \\ 0 \hfill & { otherwise} \hfill \\ \end{array} } \right.$$
